# Temporal Trends in Racial and Gender Disparities of Early Onset Colorectal Cancer in the United States: An Analysis of the CDC WONDER Database

**DOI:** 10.1007/s12029-024-01096-6

**Published:** 2024-10-01

**Authors:** Yusuf Nawras, Nooraldin Merza, Katie Beier, Aya Dakroub, Hasan Al-Obaidi, Ahmed Dheyaa Al-Obaidi, Hajera Amatul-Raheem, Eshak Bahbah, Tony Varughese, Jerome Hosny, Mona Hassan, Abdallah Kobeissy

**Affiliations:** 1 University of Toledo College of Medicine and Life Sciences, Toledo, OH USA; 2 Department of Internal Medicine, The University of Toledo, Toledo, OH USA; 3 Department of Medicine, Jamaica Hospital Medical Center, Queens, NY USA; 4 University of Baghdad College of Medicine, Baghdad, Iraq; 5 Deccan College of Medical Sciences in Hyderabad, Telangana, India; 6 Department of Internal Medicine, Al Azhar University, Cairo, Egypt; 7 Department of Internal Medicine, Hackensack University Medical Center, Hackensack, NJ USA; 8 Department of Internal Medicine, The University of Balamand, Balamand, Lebanon; 9 Department of Gastroenterology, The University of Toledo, Toledo, OH USA

**Keywords:** Early onset colorectal cancer, CDC Wonder database, Racial disparities, Gender disparities, Mortality rate, Age-adjusted mortality rate

## Abstract

**Background:**

The mortality rates of early-onset colorectal cancer (EOCRC) have surged globally over the past two decades. While the underlying reasons remain largely unknown, understanding its epidemiology is crucial to address this escalating trend. This study aimed to identify disparities potentially influencing these rates, enhancing risk assessment tools, and highlighting areas necessitating further research.

**Methods:**

Using the CDC Wide-Ranging Online Data for Epidemiologic Research (WONDER) database, this study assessed EOCRC mortality data from 2012 to 2020. Individuals under 50 years who succumbed to EOCRC were identified through the International Classification of Diseases, Tenth Revision (ICD-10) codes. Data interpretation and representation were performed using R 4.2.2 software.

**Results:**

Between 2012 and 2020, EOCRC mortality rates fluctuated marginally between 1.7 and 1.8 per 100,000. Male mortality rates increased from 1.9 to 2.0 per 100,000, while female rates varied between 1.5 and 1.6 per 100,000. Significant variations were observed across age groups, with the 40–49 years category experiencing an increase from 6.34 (2012) to 6.94 (2020) per 100,000. Racial category-based data revealed the highest mortality rates among African Americans. Geographically, Mississippi and Alabama exhibited elevated mortality rates. Age-adjusted mortality rate (AAMR) assessments indicated a marked decline for both genders from 2012 to 2020, with consistently higher rates for men.

**Conclusion:**

The findings highlight the evolving landscape of EOCRC mortality, revealing significant gender, age, and racial disparities. These results underscore the urgent need for tailored health strategies and intensified research efforts targeting these disparities.

**Supplementary Information:**

The online version contains supplementary material available at 10.1007/s12029-024-01096-6.

## Introduction

Colorectal cancer (CRC), the third most diagnosed cancer and the second leading cause of cancer-related deaths globally, poses a significant health concern [1]. Recent data from colorectal cancer statistics estimated that in 2023, there would be 153,020 new CRC cases and approximately 52,550 related deaths [2]. Over the past few decades, CRC incidence and mortality rates have witnessed a significant decline due to enhanced screening measures and treatment modalities [3]. However, a concerning shift has emerged, with a growing number of CRC diagnoses occurring in individuals under 50 years of age, known as early-onset CRC (EOCRC) [4, 5]. Currently, this younger demographic accounts for about 10% of all new CRC cases in the USA [6]. A comprehensive systemic review of 40 studies from 12 nations spanning five continents revealed an approximately 30% surge in EOCRC incidence over the past two decades [7]. This trend is significantly influenced by rising incidences in countries such as the USA, Australia, and Canada. Consequently, in 2018, the American Cancer Society (ACS) recommended that CRC screening for average-risk adults start at age 45 [8–10].

EOCRC is characterized by primarily left-sided and rectal involvement, increased mucinous and signet ring subtypes, poorer cell differentiation, a higher pathological grade, and a more advanced stage at diagnosis [11, 12]. While genetic syndromes and familial history account for about 30% of EOCRC cases, the majority seem to occur sporadically, and the underlying causes of this increase remain undetermined. Numerous risk factors have been proposed as potential contributors to EOCRC development [13, 14], including socioeconomic status, lifestyle, diet, antibiotic exposure, and intrinsic elements such as genetics, gut microbiota, and oxidative stress from early life [15]. However, the rising incidence of EOCRC cannot be entirely explained by existing CRC risk factors, suggesting that additional EOCRC risk factors may exist that have not yet been identified [15].

Several studies have highlighted significant disparities in EOCRC incidence regarding age, gender, and race. Petrick et al. found that EOCRC rates in the USA are notably higher in American Indians/Alaskan Natives and Blacks compared to Whites, with Blacks experiencing double the rates of EOCRC neuroendocrine tumors compared to Whites [16]. While overall EOCRC incidence is more prevalent in men, women have a higher incidence of distal EOCRC [16]. Additionally, racial disparities negatively impact EOCRC outcomes regardless of socioeconomic status, as demonstrated by Kamath et al. In all socioeconomic subgroups, Black patients with EOCRC had lower overall survival (OS) compared to White patients [17]. Despite these findings, available data on disparities in mortality rates among EOCRC patients is limited. Therefore, this study aimed to identify disparities potentially influencing EOCRC mortality rates, enhance risk assessment tools, and highlight areas necessitating further research. This understanding is crucial for developing targeted interventions and tailored health strategies to address the unique challenges posed by EOCRC and ultimately improve outcomes for this increasingly affected population.

## Methods

### Data Source

This study utilized the Centers for Disease Control and Prevention (CDC) Wide-Ranging Online Data for Epidemiologic Research (WONDER) database, a comprehensive online resource designed for epidemiological research [18]. Developed by the CDC, this tool provides broad access to a vast array of public health data for both professionals and the public [19]. Mortality data for the study, specifically “Underlying Cause of Death by Bridged-Race Categories” for the years 2012–2020, were retrieved and downloaded from CDC WONDER. The data included the age-adjusted mortality rate (AAMR) across different gender and racial groups in the USA through 2020. This database, compiled from death certificates from all 50 states and the District of Columbia, contains information on the primary cause of death and demographic data [19]. The aggregated statistics offered by the database include the total number of deaths and crude death rates, stratified by demographic characteristics such as age group, race, gender, race, and more.

### Study Population

This research investigated the mortality trend in EOCRC using data from 2012 to 2020. Individuals who died from EOCRC under the age of 50 were identified using the International Classification of Diseases, Tenth Edition (ICD-10) codes [refer to Supplementary material 1: Code table].

### Data Collection and Synthesis

Mortality figures and crude rates (the number of deaths per 100,000 individuals) were sourced from the CDC WONDER dataset. The data were organized for analysis using the “Group by” function available on the online interface. Data interpretation and graphical representation were conducted using R 4.2.2 software, with all illustrations generated using the ggplot2 package. This approach allowed for a detailed examination of mortality trends and disparities across different demographic groups, providing a comprehensive overview of EOCRC mortality in the USA during the study period.

### Data Privacy and Confidentiality

The study utilized publicly available data from the CDC WONDER database, which is de-identified and aggregated to protect individual privacy. No personal identifying information was accessed or used in this research, ensuring compliance with confidentiality standards and regulations.

Since the data used in this study is publicly available and de-identified, informed consent from individuals was not required. The CDC WONDER database adheres to strict data protection protocols to ensure that personal information is not disclosed.

## Results

### Crude Mortality Rates

**Fig. 1:**
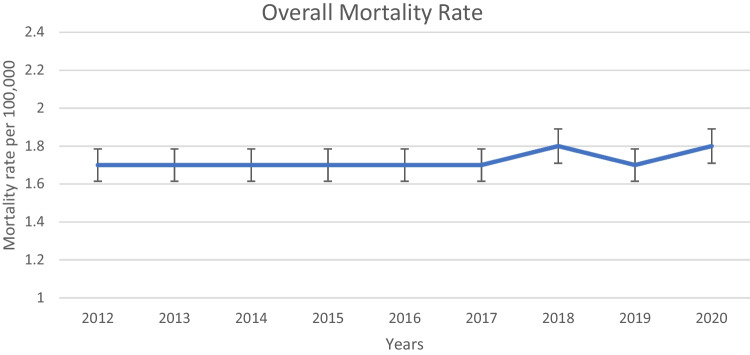
This chart illustrates the trends in the crude mortality rate of EOCRC in the USA from 2012 to 2020

The crude mortality rates for EOCRC from 2012 to 2020 are detailed in Table 1, revealing significant trends and disparities across various demographic groups. The overall crude mortality rate for EOCRC remained relatively stable over the study period, fluctuating marginally between 1.7 and 1.8 per 100,000 individuals. This stability suggests a consistent overall burden of EOCRC mortality within the U.S. population during this timeframe (Fig. 1 and Table 1).

**Table 1:** Crude mortality rate per 100,000 for the EOCRC

Crude mortality rate per 100,000									
	2012	2013	2014	2015	2016	2017	2018	2019	2020
Overall	1.7	1.7	1.7	1.7	1.7	1.7	1.8	1.7	1.8
Age, years									
20–29	0.34	0.31	0.32	0.31	0.31	0.30	0.28	0.29	0.34
30–39	1.69	1.73	1.67	1.76	1.83	1.72	1.86	1.69	1.85
40–49	6.34	6.40	6.76	6.55	6.71	6.88	6.99	6.93	6.94
Sex									
Female	1.5	1.5	1.6	1.5	1.6	1.6	1.6	1.5	1.5
Male	1.9	1.9	1.9	1.9	1.9	1.9	2.0	2.0	2.0
Race									
White	1.7	1.7	1.7	1.7	1.7	1.8	1.8	1.8	1.8
Black or African American	2	2	2.2	2	2	2	2	1.9	2.1
Asian or Pacific Islander	1.3	1.2	1.1	1.2	1.3	1.3	1.3	1.4	1.4
American India or Alaska Native	0.9	1	0.9	1.3	1.4	0.6	0.9	1.1	1.1

#### Mortality Rates by Sex

**Fig. 2:**
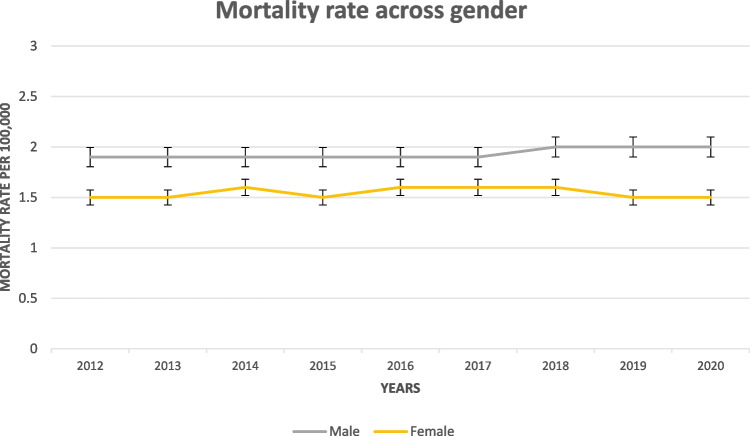
Crude mortality rate of EOCRC in the USA from 2012 to 2020, sub-grouped by gender

The mortality rate for females remained relatively constant, varying between 1.5 and 1.6 per 100,000. There was no significant change observed over the study period. The mortality rate for males was consistently higher than for females, ranging from 1.9 to 2.0 per 100,000. A slight increase was noted, from 1.9 in 2012 to 2.0 in 2018, and this rate remained steady through 2020 (Fig. 2).

#### Mortality Rates by Age Group

**Fig. 3:**
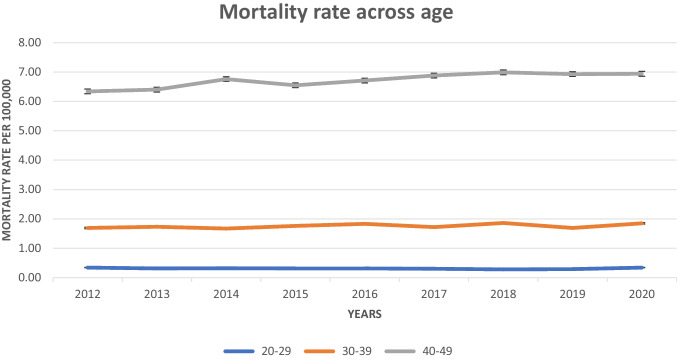
Crude mortality rate of EOCRC in the USA from 2012 to 2020, sub-grouped by age group

The mortality rate for individuals aged 20–29 years demonstrated minor fluctuations, ranging from 0.28 to 0.34 per 100,000. The rate was highest in 2012 and 2020 at 0.34, with a slight decrease observed in the intervening years, reaching its lowest point in 2018 at 0.28. For those aged 30–39 years, the mortality rate exhibited slight variability, ranging from 1.67 to 1.86 per 100,000. The highest rate occurred in 2018 at 1.86, showing a general upward trend from 2014 onwards, with a minor dip in 2019. The 40–49 age group experienced the highest mortality rates, ranging from 6.34 to 6.99 per 100,000. There was a gradual increase over the years, peaking in 2018 at 6.99, with a slight decrease to 6.94 by 2020 (Fig. 3).

#### Mortality Rates by Racial Category

The mortality rate for White individuals was stable, remaining around 1.7 to 1.8 per 100,000 throughout the study period. This group exhibited higher mortality rates compared to Whites, with rates fluctuating between 2.0 and 2.2 per 100,000. The highest rate was observed in 2014 at 2.2, with a slight decline in 2019 (1.9) before increasing again in 2020 (2.1).

**Fig. 4:**
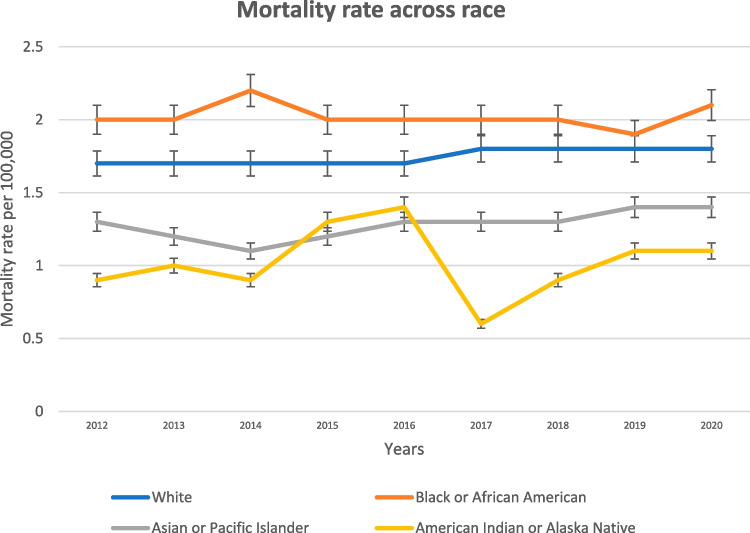
This figure illustrates the trend in the crude mortality rate of EOCRC in the USA from 2012 to 2020, with data segmented according to different racial groups

The mortality rate for Asian or Pacific Islander individuals was lower, ranging from 1.1 to 1.4 per 100,000. There was a gradual increase from 1.1 in 2014 to 1.4 in 2020. This group showed considerable variability in mortality rates, ranging from 0.6 to 1.4 per 100,000. The highest rate was in 2016 (1.4), with a notable dip in 2017 (0.6) and a gradual increase thereafter (Fig. 4).

#### Mortality Rates by Geography

**Fig. 5:**
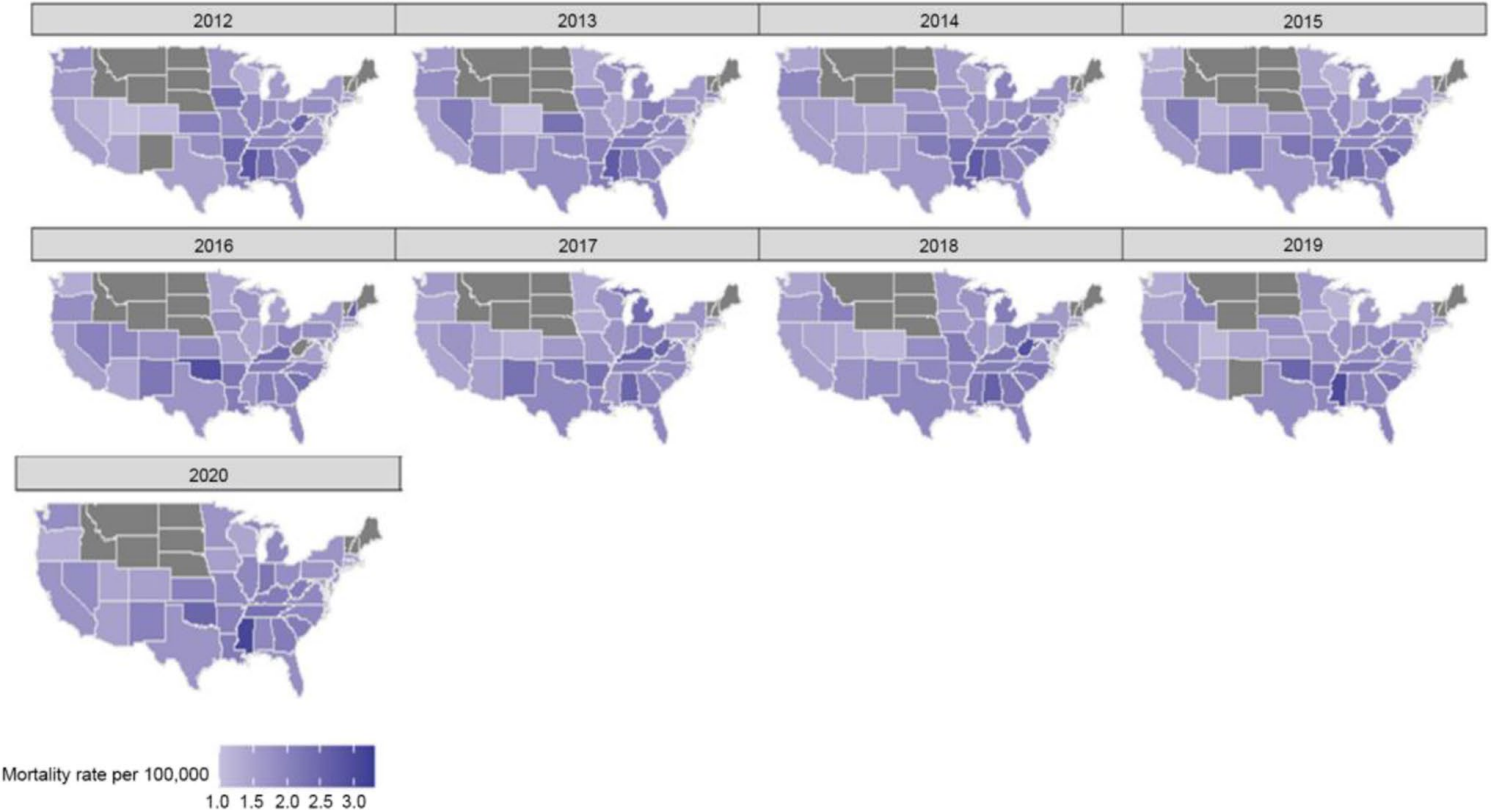
This illustrates the trend in the crude mortality rate of EOCRC in the USA from 2012 to 2020, sub-grouped by states

Mortality rates were not available for some of the states in the USA. Higher mortality rates were observed in Mississippi and Alabama, while the lowest mortality rate was observed among the Midwest regions (Fig. 5).

### AAMR by Racial Groups

Table 2 presents the AAMR for different racial groups in the USA from 2012 to 2020. Throughout the study period, Black or African American individuals consistently exhibited the highest AAMR, ranging from 18.5 to 22.3 per 100,000 population, followed by White individuals with rates ranging from 14.6 to 16.6 per 100,000. American Indian or Alaska Native and Asian or Pacific Islander groups generally showed lower AAMRs compared to Black or African American and White populations.

**Table 2:** The AAMR across different racial groups in the USA through 2020

Year	American Indian or Alaska Native	Asian or Pacific Islander	Black or African American	White
2012	13.1	12.1	22.3	16.6
2013	14.1	10.9	21.7	16.3
2014	12.1	10.6	20.6	15.9
2015	12.6	10.9	20.7	15.7
2016	11.3	10.4	20.4	15.4
2017	12.5	10.3	19.7	15.2
2018	11.4	9.8	18.8	14.9
2019	11.3	9.9	18.5	14.6
2020	11.6	10.1	18.7	14.7

### AAMR by Gender Groups

As illustrated in Table 3, there has been a notable decrease in the AAMR for both men and women over the analyzed period. In 2012, the AAMR for men stood at 20.6 (20.3–20.8), declining to 18.1 (17.9–18.3) by 2020. Likewise, for women, the AAMR decreased from 14.1 (14.0–14.3) in 2012 to 12.2 (12.1–12.4) in 2020. This longitudinal examination highlights an overall enhancement in health outcomes for both genders over time. However, despite this positive trend, the data consistently reveal higher mortality rates for men compared to women throughout the evaluated period, underscoring the necessity for further investigation into the underlying factors contributing to this disparity.

**Table 3:** The AAMR across different gender groups in the USA through 2020

	Age-adjusted mortality	
Year	Men	Women
2012	20.6 (20.3–20.8)	14.1 (14.0–14.3)
2013	20.2 (20.0–20.4)	13.8 (13.6–14.0)
2014	19.5 (19.3–19.7)	13.4 (13.3–13.6)
2015	19.2 (19.0–19.5)	13.4 (13.2–13.5)
2016	18.9 (18.7–19.1)	13.0 (12.9–13.2)
2017	18.5 (18.3–18.7)	12.8 (12.7–13.0)
2018	18.3 (18.1–18.5)	12.5 (12.3–12.6)
2019	17.8 (17.6–18.0)	12.2 (12.1–12.4)
2020	18.1 (17.9–18.3)	12.2 (12.1–12.4)

## Discussion

This study investigated racial, gender and geographical disparities in EOCRC in the US, using data from the CDC WONDER database. This comprehensive resource provided extensive information on EOCRC mortality rates from 2012 to 2020, allowing for a robust analysis of trends and disparities across demographic groups. Our findings indicated that the overall mortality rate for EOCRC has remained relatively stable over the past decade, with a minor increase from 1.7 per 100,000 individuals in 2012 to 1.8 per 100,000 individuals in 2020. This stability is consistent with existing literature that suggests an escalating incidence of EOCRC, especially in high-income countries [20, 21]. The constancy in mortality rates aligns with studies suggesting that advancements in screening, diagnosis, and management of EOCRC might be contributing factors [22–24].

Significant disparities were evident when data were examined according to gender, age, and racial category. Men with EOCRC consistently exhibited higher mortality rates than women. This observation is consistent with prior studies, such as Rawla et al., which documented elevated colorectal cancer incidence and mortality rates in males [3]. Such findings suggest that men may inherently face higher CRC susceptibility and a less favorable prognosis, potentially stemming from an interplay of biological, environmental, and lifestyle determinants [3, 25]. The underlying reasons behind this persistent gender disparity in EOCRC mortality remain unclear, highlighting the need for deeper exploration.

A dramatic rise in the mortality rate was observed among the age group of 20 to 50 years, particularly in the 40–49 years category. This supports the current recommendation by the American Cancer Society (ACS) to lower the starting age for average-risk adults to 45 years for CRC screening [9, 26]. Timely screening has been proven to be an effective strategy in reducing mortality associated with CRC [27, 28]. Therefore, increasing awareness and advocating for early and regular screening could help curb the rising mortality in this age group [29].

Racial disparities were also prominent in our study. African Americans were found to have the highest EOCRC mortality rate compared to other ethnic groups. This is consistent with previous research that has highlighted racial disparities in CRC incidence and outcomes [30]. This racial disparity could be attributed to a multitude of factors, including socio-economic status, access to healthcare, health behavior, and potential genetic predispositions [17]. Efforts should be directed to reduce this racial disparity through the implementation of targeted public health initiatives and policies that promote equal access to screening and healthcare services.

Our study also found geographical disparities in EOCRC mortality across different states. Higher mortality rates were observed in Mississippi and Alabama, while the Midwest regions recorded lower mortality rates. Previous studies have reported similar geographic disparities, which might be influenced by regional differences in healthcare access, environmental factors, socio-economic status, and lifestyle habits [31].

## Strengths and Limitations

One of the key strengths of this study is the utilization of the CDC WONDER database, which is a reliable and comprehensive source of mortality data. The long study period (2012–2020) allows for a thorough examination of trends over time. Additionally, the study’s stratification of data by age, sex, and race provides a detailed understanding of the disparities in EOCRC mortality, highlighting areas in need of targeted interventions. Despite its strengths, the study has several limitations. The descriptive nature of the analysis and potential underestimation of mortality rates in the CDC WONDER database, which relies on the accurate reporting and coding of death certificates, are notable constraints. Additionally, our study focused solely on mortality rates and did not consider the incidence or survival rates of EOCRC. Due to the suppression criteria of the CDC WONDER, death counts under 10 were not accessible from the dataset, potentially influencing our results.

## Future Directions

The findings of this EOCRC study sound a critical alarm, highlighting the urgent need for action on several aspects. With men consistently facing higher mortality rates, it is important to target research efforts toward gender-specific determinants of the disease and tailor interventions accordingly. Racial disparities necessitate immediate actions and modifications in healthcare policies to ensure equitable healthcare access across all racial and ethnic groups. The surge in mortality rates among those aged 40–49 also demands a prompt reassessment of CRC screening guidelines. Moreover, geographical disparities highlight the pressing need for region-specific healthcare strategies, taking into account unique local factors affecting health outcomes. Finally, the healthcare community, policymakers, and stakeholders must collaborate together, translating these findings into concrete, targeted actions that tackle EOCRC disparities head-on.

## Conclusion

In conclusion, our study highlights disparities in EOCRC mortality trends in the USA. While overall mortality rates remained stable, significant disparities by gender, age, race, and geography were evident. These findings highlight the urgent need for targeted interventions to address these disparities. Addressing these disparities through targeted public health strategies, policy changes, and increased awareness can lead to better outcomes and reduce the burden of EOCRC in the USA. Future research should focus on the factors driving these disparities, with an emphasis on the roles of genetics, lifestyle, and environmental factors.

## Supplementary Information

Below is the link to the electronic supplementary material.

Supplementary file1 (DOCX 13.0 KB)

## Data Availability

No datasets were generated or analysed during the current study.

## Abbreviations

EOCRC: Early Onset Colorectal Cancer
CRC: Colorectal cancer
CDC-WONDER: Wide-ranging Online Data for Epidemiologic Research (WONDER), for Disease Control and Prevention’s (CDC)
ACS: American Cancer Society
ICD-10 codes: International Classification of Disease, the tenth revision
AAMR: Age-adjusted mortality rate

## References

[1] World Health Organization (WHO). Colorectal cancer: key facts [Internet]. 2023. Available from https://www.who.int/news-room/fact-sheets/detail/colorectal-cancer#:~:text=Colon%20cancer%20is%20the%20second,and%20mortality%20rates%20were%20observed.

[2] Siegel RL, Wagle NS, Cercek A, Smith RA, Jemal A. Colorectal cancer statistics, 2023. CA Cancer J Clin. 2023;73(3):233–54. 36856579 10.3322/caac.21772

[3] Rawla P, Sunkara T, Barsouk A. Epidemiology of colorectal cancer: incidence, mortality, survival, and risk factors. Gastroenterol Rev [Internet]. 2019;14(2):89–103. 10.5114/pg.2018.81072. 10.5114/pg.2018.81072 PMC6791134 31616522

[4] AlZaabi A, AlHarrasi A, AlMusalami A, AlMahyijari N, Al Hinai K, ALAdawi H, et al. Early onset colorectal cancer: challenges across the cancer care continuum. Ann Med Surg. 2022;82:104453. 10.1016/j.amsu.2022.104453 PMC9577444 36268309

[5] Wu CW-K, Lui RN. Early-onset colorectal cancer: current insights and future directions. World J Gastrointest Oncol. 2022;14(1):230–41. 35116113 10.4251/wjgo.v14.i1.230 PMC8790420

[6] Stoffel EM, Murphy CC. Epidemiology and mechanisms of the increasing incidence of colon and rectal cancers in young adults. Gastroenterology. 2020;158(2):341–53. 31394082 10.1053/j.gastro.2019.07.055 PMC6957715

[7] Saad El Din K, Loree JM, Sayre EC, Gill S, Brown CJ, Dau H, et al. Trends in the epidemiology of young-onset colorectal cancer: a worldwide systematic review. BMC Cancer. 2020;20(1):288. 32252672 10.1186/s12885-020-06766-9 PMC7137305

[8] Abualkhair WH, Zhou M, Ahnen D, Yu Q, Wu X-C, Karlitz JJ. Trends in incidence of early-onset colorectal cancer in the united states among those approaching screening age. JAMA Netw Open. 2020;3(1):e1920407. 32003823 10.1001/jamanetworkopen.2019.20407 PMC7042874

[9] Wolf AMD, Fontham ETH, Church TR, Flowers CR, Guerra CE, LaMonte SJ, et al. Colorectal cancer screening for average-risk adults: 2018 guideline update from the American Cancer Society. CA Cancer J Clin. 2018;68(4):250–81. 29846947 10.3322/caac.21457

[10] Peterse EFP, Meester RGS, Siegel RL, Chen JC, Dwyer A, Ahnen DJ, et al. The impact of the rising colorectal cancer incidence in young adults on the optimal age to start screening: microsimulation analysis I to inform the American Cancer Society colorectal cancer screening guideline. Cancer. 2018;124(14):2964–73. 29846933 10.1002/cncr.31543 PMC6033623

[11] Álvaro E, Cano JM, García JL, Brandáriz L, Olmedillas-López S, Arriba M, et al. Clinical and molecular comparative study of colorectal cancer based on age-of-onset and tumor location: two main criteria for subclassifying colorectal cancer. Int J Mol Sci. 2019;20(4):968. 30813366 10.3390/ijms20040968 PMC6413061

[12] Vuik FER, Nieuwenburg SAV, Nagtegaal ID, Kuipers EJ, Spaander MCW. Clinicopathological characteristics of early onset colorectal cancer. Aliment Pharmacol Ther. 2021;54(11–12):1463–71. 34637541 10.1111/apt.16638 PMC9292775

[13] Medici B, Riccò B, Caffari E, Zaniboni S, Salati M, Spallanzani A, et al. Early onset metastatic colorectal cancer: current insights and clinical management of a rising condition. Cancers (Basel). 2023;15(13):3509. 37444619 10.3390/cancers15133509 PMC10341111

[14] Ullah F, Pillai AB, Omar N, Dima D, Harichand S. Early-onset colorectal cancer: current insights Cancers (Basel). 2023;15(12):3202. 37370811 10.3390/cancers15123202 PMC10296149

[15] Hofseth LJ, Hebert JR, Chanda A, Chen H, Love BL, Pena MM, et al. Early-onset colorectal cancer: initial clues and current views. Nat Rev Gastroenterol Hepatol. 2020;17(6):352–64. 32086499 10.1038/s41575-019-0253-4 PMC10711686

[16] Petrick JL, Barber LE, Warren Andersen S, Florio AA, Palmer JR, Rosenberg L. Racial disparities and sex differences in early- and late-onset colorectal cancer incidence, 2001–2018. Front Oncol. 2021;11:734998. 34568072 10.3389/fonc.2021.734998 PMC8459723

[17] Kamath SD, Torrejon N, Wei W, Tullio K, Nair KG, Liska D, et al. Racial disparities negatively impact outcomes in early-onset colorectal cancer independent of socioeconomic status. Cancer Med [Internet]. 2021;10(21):7542–50. 10.1002/cam4.4276. 34647438 10.1002/cam4.4276 PMC8559495

[18] Friede A, Reid JA, Ory HW. CDC WONDER: a comprehensive on-line public health information system of the Centers for Disease Control and Prevention. Am J Public Health. 1993;83(9):1289–94. 8395776 10.2105/ajph.83.9.1289 PMC1694976

[19] Centers for Disease Control and Prevention. CDC WONDER (wide-ranging online data for epidemiologic research) database [Internet]. 2022. Available from https://wonder.cdc.gov/.

[20] Araghi M, Soerjomataram I, Bardot A, Ferlay J, Cabasag CJ, Morrison DS, et al. Changes in colorectal cancer incidence in seven high-income countries: a population-based study. Lancet Gastroenterol Hepatol. 2019;4(7):511–8. 31105047 10.1016/S2468-1253(19)30147-5 PMC7617144

[21] Loomans-Kropp HA, Umar A. Increasing incidence of colorectal cancer in young adults. J Cancer Epidemiol. 2019;2019:9841295. 31827515 10.1155/2019/9841295 PMC6885269

[22] Dharwadkar P, Zaki TA, Murphy CC. Colorectal cancer in younger adults. Hematol Oncol Clin North Am. 2022;36(3):449–70. 35577711 10.1016/j.hoc.2022.02.005 PMC9177054

[23] Murphy CC, Sandler RS, Sanoff HK, Yang YC, Lund JL, Baron JA. Decrease in incidence of colorectal cancer among individuals 50 years or older after recommendations for population-based screening. Clin Gastroenterol Hepatol Off Clin Pract J Am Gastroenterol Assoc. 2017;15(6):903–9.e6. 10.1016/j.cgh.2016.08.037 PMC5337450 27609707

[24] Siegel RL, Miller KD, Jemal A. Colorectal cancer mortality rates in adults aged 20 to 54 years in the United States, 1970–2014. JAMA. 2017;318:572–4. 28787497 10.1001/jama.2017.7630 PMC5817468

[25] Carethers JM. Screening for colorectal cancer in African Americans: determinants and rationale for an earlier age to commence screening. Dig Dis Sci. 2015;60(3):711–21. 25540085 10.1007/s10620-014-3443-5 PMC4369177

[26] Shaukat A, Kahi CJ, Burke CA, Rabeneck L, Sauer BG, Rex DK. ACG clinical guidelines: colorectal cancer screening 2021. Am J Gastroenterol. 2021;116(3):458–79. 33657038 10.14309/ajg.0000000000001122

[27] Li D. Recent advances in colorectal cancer screening. Chronic Dis Transl Med. 2018;4:139–47. 30276360 10.1016/j.cdtm.2018.08.004 PMC6160607

[28] Brenner AT, Dougherty M, Reuland DS. Colorectal cancer screening in average risk patients. Med Clin North Am. 2017;101(4):755–67. 28577625 10.1016/j.mcna.2017.03.007 PMC6376481

[29] Murphy CC, Wallace K, Sandler RS, Baron JA. Racial disparities in incidence of young-onset colorectal cancer and patient survival. Gastroenterology. 2019;156(4):958–65. 30521807 10.1053/j.gastro.2018.11.060 PMC6409160

[30] DeSantis CE, Siegel RL, Sauer AG, Miller KD, Fedewa SA, Alcaraz KI, et al. Cancer statistics for African Americans, 2016: progress and opportunities in reducing racial disparities. CA Cancer J Clin. 2016;66(4):290–308. 26910411 10.3322/caac.21340

[31] Siegel RL, Miller KD, Jemal A. Cancer statistics, 2018. CA Cancer J Clin [Internet]. 2018;68(1):7–30. 10.3322/caac.21442. 29313949 10.3322/caac.21442

