# Supplementary material for: Temporal Trends in Racial and Gender Disparities of Early Onset Colorectal Cancer in the United States: An Analysis of the CDC WONDER Database
Source: J Gastrointest Cancer. 2024 Oct 1;55(4):1511–9. doi: 10.1007/s12029-024-01096-6 (PMC11464567; doi:10.1007/s12029-024-01096-6)
Supplement: Supplementary file 1 — Supplementary file1 (DOCX 13.0 KB) [file 12029_2024_1096_MOESM1_ESM.docx]

**International Classification of Disease, the tenth revision (ICD-10) codes:**

C18.0, C18.1, C18.2, C18.3, C18.4, C18.5, C18.6, C18.7, C18.8, C18.9, C19, C20, C21.1, C21.2, C21.8.
